# Efficacy and safety of Qingre Huatan Formula for the prevention of early neurological deterioration in patients with acute ischemic stroke (QUIET): rationale and design for a randomized double-blind placebo-controlled study

**DOI:** 10.3389/fmed.2026.1754648

**Published:** 2026-04-13

**Authors:** Xiangyi Zheng, Zhaowen Yang, Huihong Wu, Miaomiao Zhao, Cuilin Que, Xuejiao Xiong, Yulin Mu, Genming Zhang, Ligaoge Kang, Ying Gao, Xinxing Lai

**Affiliations:** 1 Department of Neurology, Dongzhimen Hospital, Beijing University of Chinese Medicine, Beijing, China; 2 Fangshan Hospital, Beijing University of Chinese Medicine, Beijing, China; 3 Institute for Brain Disorders, Beijing University of Chinese Medicine, Beijing, China

**Keywords:** acute ischemic stroke, protocol, Qingre Huatan Formula, randomized controlled trial, statistical analysis plan, traditional Chinese medicine

## Abstract

**Background:**

Early neurological deterioration (END) remains challenging despite guideline-based treatments in patients with acute ischemic stroke (AIS). To date, limited evidence has been established to prevent END. Therefore, this study aims to determine whether Qingre Huatan Formula (QHF) initiated within 48 h of stroke onset prevents END in AIS patients compared with placebo.

**Methods:**

The Qingre Huatan Formula for the Prevention of Early Neurological Deterioration in Acute Ischemic Stroke (QUIET) trial is a randomized, placebo-controlled, double-blind, parallel-group pilot study. Seventy-two eligible patients with AIS within 48 h of symptom onset will be randomly (1:1) assigned to receive either QHF or placebo treatment for 10 days and will subsequently be followed up to 90-days. The primary outcome is the proportion of END within 7 days of stroke onset, defined as a National Institutes of Health Stroke Scale (NIHSS) score increase of ≥2 points. Secondary outcomes mainly include the change in NIHSS score from baseline to 10 days after randomization, and the proportion of patients achieving a good functional outcome (modified Rankin Scale score ≤ 2).

**Discussion:**

This trial will provide valuable evidence for the efficacy and safety of traditional Chinese medicine for END in AIS patients.

**Clinical trial registration:**

https://register.clinicaltrials.gov, identifier NCT06857487 (registered on February 27, 2025).

## Introduction

Stroke is a major global health threat, imposing a substantial burden on individuals and public health systems (1, 2). Ischemic stroke constitutes roughly two-thirds of all new stroke events worldwide (3). Acute ischemic stroke (AIS) is marked by abrupt neurological deficit onset and rapid clinical progression, often culminating in severe disability. Despite guideline-based usual care, however, 5%−40% of patients develop early neurological deterioration (END) within hours or days of symptom onset (4), which is associated with poor clinical outcomes and increased mortality (4–7).

END is one of the most significant challenges in AIS management. Currently, its clinical handling faces three core dilemmas: First, its pathogenesis involves multiple pathophysiological pathways that are intrinsically complex and accompanied by numerous risk factors (8). Second, validated early prediction tools are lacking, impeding rapid identification of high-risk END patients shortly after onset and thereby delaying intensified monitoring and individualized intervention. Moreover, current reperfusion, antithrombotic and neuroprotective strategies show limited efficacy specifically targeting END (9). As a result, the development of safe and effective therapies for this condition has become an urgent unmet need in the acute management of ischemic stroke.

Traditional Chinese medicine (TCM) has accumulated substantial experience in syndrome differentiation and proven herbal formulas for the prevention and treatment of AIS. High-quality clinical evidence has recently emerged, with studies published in leading international journals, underscoring TCM's pivotal role in this field and its encouraging prospects for curbing END (10–12). Our previous work identified phlegm-heat syndrome as a core pattern in the acute stage of ischemic stroke (13). Moreover, the persistence of phlegm-heat syndrome slows neurological recovery and increases the risk of END (14). These findings indicate that prompt administration of heat-clearing and phlegm-resolving therapy is critical for resolving phlegm-heat syndrome, thereby promoting neurological recovery and preventing END. Accordingly, we developed the Qingre Huatan Formula (QHF), a traditional Chinese medicine formula designed specifically for clearing heat and resolving phlegm in AIS patients. Therefore, we designed a randomized, placebo-controlled, double-blind, parallel-group pilot study to demonstrate the efficacy and safety of QHF for the prevention of END in AIS patients.

## Methods

### Study design

The Qingre Huatan Formula for the prevention of early neurological deterioration in acute ischemic stroke (QUIET) trial is a randomized, placebo-controlled, double-blind, parallel-group pilot study registered with ClinicalTrials.gov (NCT06857487) prior to patient enrollment. The primary objective of this study is to assess the effectiveness and safety of QHF vs. placebo for AIS patients at high risk of END. We hypothesize that QHF will be superior to placebo at reducing the risk of END within 7 days of stroke onset, defined as a National Institutes of Health Stroke Scale (NIHSS) score increase of ≥2 points. Furthermore, we will explore the neuroimaging, biological indicators and other dimensions of patients suffering from the phlegm-heat syndrome in AIS. This trial protocol was approved by the institutional review board of Dongzhimen Hospital, Beijing University of Chinese Medicine, Beijing, China (No. 2023DZMEC-410-02). The protocol design is based on the Standard Protocol Items: Recommendations for Interventional Trials (SPIRIT) guidelines (15). The SPIRIT checklist is shown in the Supplementary material.

### Recruitment of participants

**Figure 1:**
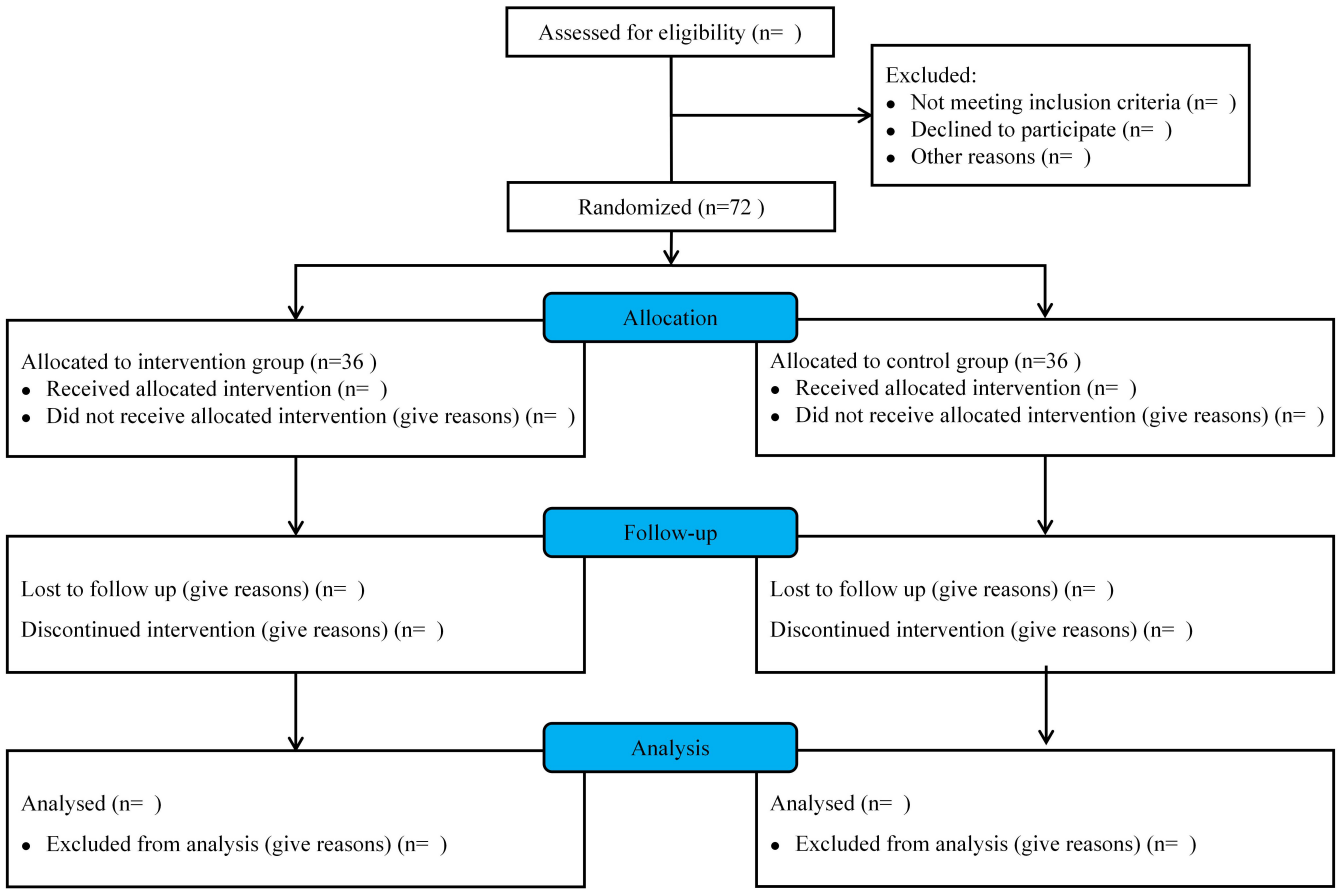
The diagram of the QUIET trial.

This study will recruit 72 patients diagnosed with AIS. Open recruitment for AIS patients will be performed both in Dongzhimen Hospital of Beijing University of Chinese Medicine (Beijing, China) and Fangshan Hospital of Beijing University of Chinese Medicine (Beijing, China). Recruitment advertisements will be displayed online and on hospital notice boards, allowing patients to voluntarily contact investigators. Potential patients will be screened for eligibility based on the inclusion and exclusion criteria. Eligible patients will be informed of the risks and benefits of the study. If they agree to participate in the study, patients and/or their representatives will sign the informed consent form. Patient enrollment will begin in December 2025 and end in October 2026. The schematic diagram of the patient timeline is presented in Figure 1.

### Patient selection

This study will enroll patients aged 18–80 years who are diagnosed with AIS within 48 h of symptom onset and have a total score ≥10 for both TCM phlegm-dampness and internal-fire syndrome elements, as assessed by the Ischemic Stroke TCM Syndrome Diagnostic Scale (ISDS). The ISDS is a validated instrument developed by our research team (16); the full scale and detailed scoring rules are provided in the Supplementary material. Syndrome differentiation will be performed by at least two attending physicians or higher who have received standardized training on the ISDS. In cases of disagreement, a third senior expert will be consulted to reach a consensus. Inter-rater reliability will be assessed using the Kappa statistic based on 20 pilot cases, with a target Kappa value ≥0.80 indicating good consistency. Regular quality control meetings will be held throughout the trial to ensure consistency in syndrome differentiation.

Patients must also meet the criteria for being at high risk of END, defined as either: (1) MRI showing new ischemic lesions with a DWI-ASPECTS score ≤ 7; or (2) at least two of the following clinically established risk factors for END: hypertension, diabetes mellitus, atrial fibrillation, hyperglycemia at admission, baseline NIHSS score, large vessel occlusion (internal carotid artery or middle cerebral artery M1 segment), or elevated peripheral inflammatory markers. These criteria are based on current evidence and guidelines for END prediction (5, 17–19). The detailed inclusion and exclusion criteria are provided in Table 1.

**Table 1:** Inclusion and exclusion of the QUIET trial.

Inclusion criteria
1. Inpatients diagnosed with AIS.
2. Patients diagnosed with phlegm-heat syndrome based on the Ischemic Stroke TCM Syndrome Diagnostic Scale (ISDS).
3. Patients at high risk of END, defined as either: (1) MRI showing new ischemic lesions with a DWI-ASPECTS score ≤ 7; or (2) at least two of the following clinically established risk factors for END: hypertension, diabetes mellitus, atrial fibrillation, hyperglycemia at admission, baseline NIHSS score, large vessel occlusion (internal carotid artery or middle cerebral artery M1 segment), or elevated peripheral inflammatory markers.
4. Acute ischemic stroke within 48 hours after symptom onset (interval from symptom onset to eligibility screening and enrollment)
5. Aged 18–80 years, male or female.
6. The patient or their legal representative has signed the informed consent form.
15.6-7.4,13.3499pt**Exclusion criteria**
1. Suspected secondary stroke induced by brain tumor, traumatic brain injury, or hematological disorders.
2. Dependent on activities of daily living before the present acute stroke (defined as pre-stroke mRS score ≥ 2).
3. Motor dysfunction caused by other severe diseases (e.g., severe osteoarthritis).
4. Known severe liver dysfunction (ALT/AST > 2 × ULN).
5. Known severe kidney dysfunction (serum creatinine > 1.5 × ULN).
6. Known severe aphasia or mental illness affecting clinical information collection and evaluation.
7. Known hypersensitivity to any component of Qingre Huatan Formula or Chinese herbal medicines with the same efficacy.
8. Pregnancy, potential pregnancy or breastfeeding.
9. Currently participating in other interventional clinical trials.

AIS, acute ischemic stroke; ALT, alanine aminotransferase; ASPECTS, Alberta Stroke Program Early CT Score; AST, aspartate transaminase; DWI, diffusion weighted imaging; END, early neurological deterioration; MRI, magnetic resonance imaging; mRS, Modified Rankin Scale; TCM, traditional Chinese medicine; ULN, upper limit of normal value.

### Randomization, allocation, and blinding

Eligible patients will be randomly assigned (1:1) to the intervention or control group using permuted block randomization with a block size of 4, stratified by centre. The allocation sequence, generated and kept under confidential seal by an independent biostatistician using the SAS 9.4 PROCPLAN, will simultaneously serve as the drug-code list for labeling the identical packages of trial medication and matched placebo.

To maintain blinding, QHF and placebo granules are identical in appearance, taste, smell, and weight, and are produced by New Green Pharmaceutical Co., Ltd. All investigators, patients, statisticians, and outcome assessors will remain blinded to group assignment throughout the trial until the blind codes are unlocked. All adverse events will be reported using standardized terminology without revealing group assignment, and concomitant medications with potential unblinding effects (e.g., TCM treatments with similar efficacy) are strictly prohibited. Regular monitoring will assess blinding integrity, with any breaches documented and reported.

Emergency unblinding will be permitted only for serious adverse events (SAEs) where knowledge of treatment assignment is essential for patient management. Requests must be approved by the principal investigator or an independent Data Safety Monitoring Board (DSMB) member and executed via a secure web-based system or sealed envelope. All unblinding events will be documented, and affected participants will be excluded from per-protocol analyses while others remain blinded.

### Treatment

Eligible patients will be randomly assigned to either the intervention group or the control group. The intervention group will receive QHF for 10 days, and the control group will receive a QHF placebo. QHF is a Chinese herbal compound medicine. The composition of one dose of QHF is provided in the Supplementary material. The main ingredients of QHF placebo include caramel, picrin and burnt grain buds. In this study, concentrated Chinese herbal medicine granules will be used to replicate the traditional method of preparing herbal tonics. All QHF and placebo granules will be produced and packed by New Green Pharmaceutical Co., Ltd., Sichuan, People's Republic of China, with identical color, smell, and weight.

To ensure batch-to-batch consistency, all raw herbs used in QHF are authenticated and comply with the quality standards specified in the Chinese Pharmacopoeia (2020 edition). The manufacturing process is conducted under good manufacturing practice (GMP) conditions by New Green Pharmaceutical Co., Ltd. (Sichuan, China). A certificate of analysis is issued for each production batch.

Chemical fingerprinting of QHF has been established using high-performance liquid chromatography (HPLC). Four representative batches were analyzed, and 18 common peaks were identified in the fingerprint. The similarity between batches was calculated using the Chromatographic Fingerprint Similarity Evaluation Software for traditional Chinese medicine (2012 Edition), and all batches showed a similarity of ≥0.90 compared to the reference fingerprint. Key peaks corresponding to marker compounds such as geniposide, salvianolic acid B, and rhein have been annotated. Detailed fingerprinting data, including chromatograms and similarity results, are provided in the Supplementary material.

**Figure 2:**
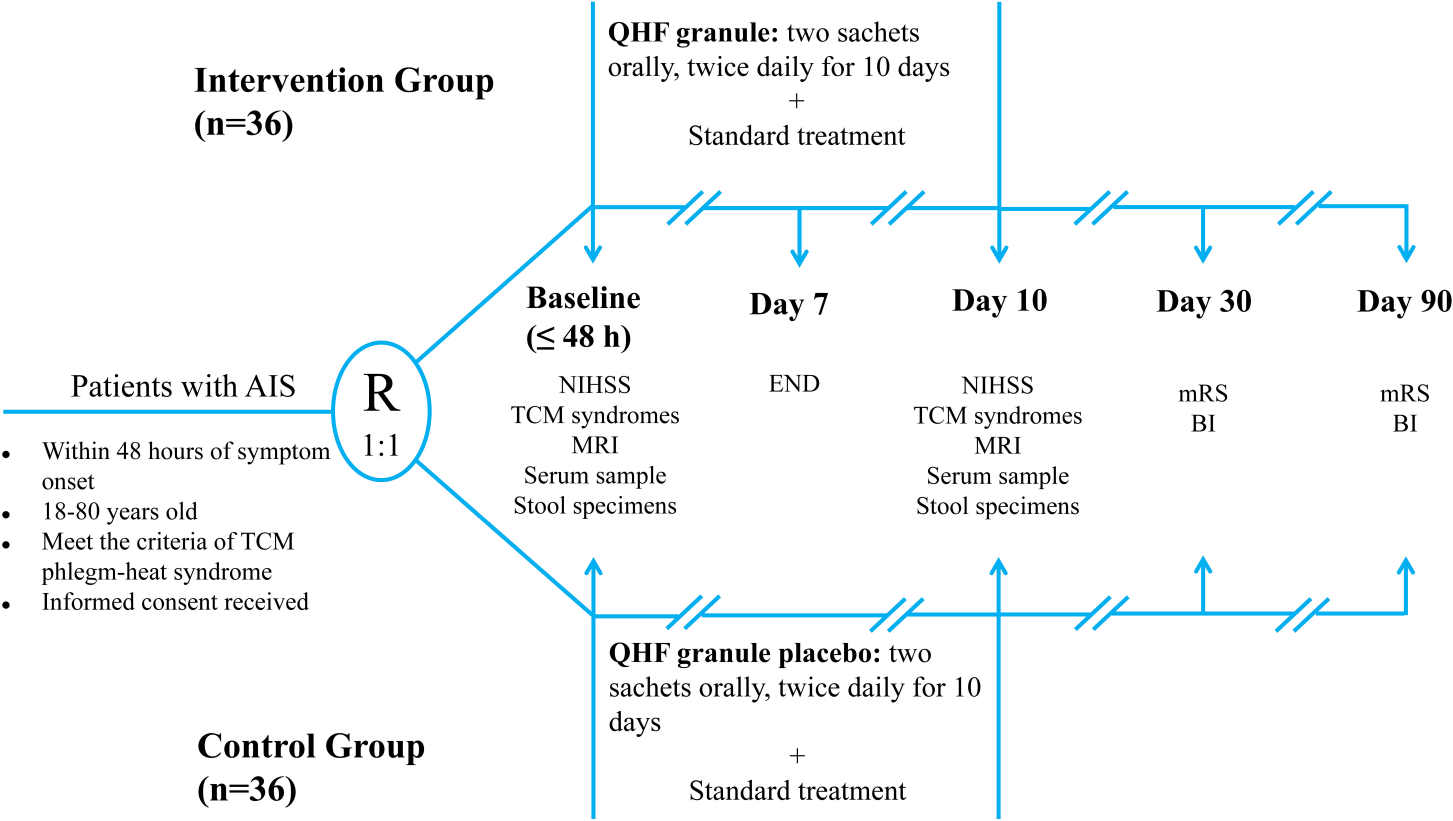
The flowchart of the QUIET trial. AIS, acute ischemic stroke; BI, Barthel Index; END, early neurological deterioration; MRI, magnetic resonance imaging; mRS, Modified Rankin Scale; NIHSS, National Institutes of Health Stroke Scale; QHF, Qingre Huatan Formula; TCM, traditional Chinese medicine.

Each dose of the study granules (QHF or matching placebo) is packaged in four sachets. Participants will take two sachets orally, twice daily, approximately 30 min after breakfast and dinner. At the same time, all patients will receive guideline-based standard care for AIS, including the anti-platelet therapy, the comprehensive management of vascular risk factors, and early initiation of multidisciplinary rehabilitation (9). Figure 2 presents the flowchart of the QUIET study. Any medication sharing composition or efficacy with the study drug is prohibited throughout the trial, including TCM decoctions, TCM granules, and Chinese patent medicines. During the observation period, the name, type, dose, and duration of all concomitant medications should be recorded in the Case Report Forms (CRFs). The study drug will be discontinued in case of SAEs occurrences, study withdrawal request from patients or their representatives, or poor compliance or non-adherence to the prescribed interventions. All reasons for stopping interventions will be recorded faithfully.

### Efficacy outcomes

The primary outcome is the proportion of END within 7 days of stroke onset, defined as an NIHSS score increase of ≥2 points compared with baseline. Secondary outcomes are as follows: (1) change in neurological deficit from baseline to 10 days after randomization; (2) the proportion of patients achieving a good functional outcome, defined as a modified Rankin Scale (mRS) score ≤ 2 on day 30 and 90; (3) activities of daily living measured by Barthel Index (BI) on day 30 and 90.

### Exploratory outcomes

#### Biological outcomes

We will compare between-group changes in intercellular adhesion molecule-1 (ICAM-1), interleukin-6 (IL-6), tumor necrosis factor-α (TNF-α), matrix metalloproteinase-9 (MMP-9) and serum neurofilament light chain (sNfL) levels from baseline to 10 days after randomization. In addition, intestinal microbiota will be profiled by metagenomic sequencing of stool specimens collected at baseline and 10 days after randomization.

#### Neuroimaging outcomes

Infarct volume and the extent of cerebral edema will be quantified using DWI and FLAIR sequences on MRI obtained at baseline and 10 days after randomization.

#### Safety outcomes

Safety will be monitored continuously throughout the trial, capturing all adverse events (AEs), treatment-emergent adverse events (TEAEs), SAEs, and clinically relevant changes in vital signs, physical examination, ECG, and standard laboratory parameters.

An adverse event (AE) is defined as any untoward medical occurrence that emerges or worsens during the study period, from informed consent through final follow-up. AEs will be graded according to the common terminology criteria for adverse events (CTCAE) Version 5.0 as mild (grade 1), moderate (grade 2), severe (grade 3), life-threatening (grade 4), or fatal (grade 5). treatment-emergent adverse events (TEAEs) are AEs that occur or worsen following the first dose of study medication.

Serious adverse events (SAEs) are defined as any AE that,s regardless of dose or causality: (1) results in death; (2) is life-threatening; (3) requires inpatient hospitalization or prolongation of existing hospitalization; (4) results in persistent or significant disability or incapacity; (5) is a congenital anomaly or birth defect; or (6) requires intervention to prevent permanent impairment or damage.

At each study visit, participants will be systematically questioned about any AEs using non-leading questions. All AEs, regardless of severity or causality, will be recorded in CRFs with detailed information including onset date, duration, severity, relationship to study drug, actions taken, and outcome. The relationship between the AE and the study drug will be assessed by the site investigator using a five-category scale: definitely related, probably related, possibly related, probably unrelated, or definitely unrelated. All AEs will be managed according to standard clinical practice, with appropriate treatment provided as necessary.

All SAEs must be reported to the principal investigator within 24 h of awareness. The principal investigator will then report the SAEs to the institutional review board (IRB) and the DSMB within the required timeframe (typically within 7 calendar days for fatal or life-threatening events, and within 15 calendar days for other SAEs). All SAEs will be followed until resolution or stabilization.

The overall schedule of trial-related activities and data collection is presented in Table 2.

**Table 2:** Schedule of the study process.

Time point	Treatment period					Follow-up period	
	Baseline (Day 1)	Day 3	Day 5	Day 7	Day 10 or discharge	Day 30	Day 90
Eligibility screening	×						
Informed consent	×						
Demographics	×						
Medical history	×						
Vital signs	×				×		
Physical examination	×				×		
Neurological evaluation	×	×	×	×	×		
CT/MRI	×				×		
ECG	×				×		
Laboratory examination	×				×		
Allocation	×						
TCM syndromes	×			×	×		
NIHSS	×	×	×	×	×		
mRS						×	×
BI						×	×
Serum sample	×				×		
Stool sample	×				×		
Concomitant medications					×	×	×
Complications	×	×		×	×		
AEs and SAEs	×	×	×	×	×	×	×

AE, adverse event; BI, Barthel Index; CT, computed tomography scan; ECG, electrocardiogram; MRI, magnetic resonance imaging; mRS, Modified Rankin Scale; NIHSS, National Institutes of Health Stroke Scale; SAE, serious adverse event; TCM, traditional Chinese medicine.

### Follow up procedures

All follow-up assessments will be performed by independent interviewers trained in neurology who are certified according to a standard operating procedure (SOP) and remain fully blinded to treatment allocation throughout the trial. Structured telephone interviews will be conducted standardized CRF forms at 30 and 90 days after symptom onset. The CRFs include mRS and BI scores, stroke recurrence, new-onset vascular events, medication use, and rehabilitation services received during the follow-up period. All interviews will be recorded and saved.

NIHSS assessments will be performed by certified neurologists or neurology residents who have completed standardized NIHSS training. All assessors will be blinded to treatment allocation. Prior to study initiation, assessors will be required to achieve ≥90% agreement with expert consensus scores across 10 validation cases. Throughout the trial, 10% of assessments will be randomly audited by an independent expert, and periodic refresher training will be conducted to maintain inter-rater reliability.

### Data management and quality assurance

The study implements a rigorous data management system with multiple quality control measures. All clinical investigators and research coordinators will receive standardized training on the trial operating procedures before patient recruitment, covering data collection, proper CRF completion, outcome assessment, and serum sample collection. Researchers will verify all CRF entries against source documents for consistency, with modifications clearly documented using lined-through corrections accompanied by signatures and dates.

A critical quality control measure involves the independent double-data-entry system where two separate personnel input the same data to minimize errors. The database then performs automated range and logic checks to identify discrepancies. For quality assurance, regular on-site monitoring visits will be conducted by clinical research associates who will perform source data verification by sampling original records against CRF entries. The monitoring team will also evaluate protocol compliance across all study sites, with findings communicated through monitoring reports and study newsletters to maintain consistent standards.

All research data will be stored securely with multiple backups on different media, while original CRFs will be archived sequentially with a retrieval index. Throughout the study period, investigators will make concerted efforts to maintain patient follow-up. All patient-related information will be stored in locked file cabinets and identified only by coded numbers to protect confidentiality. These comprehensive measures aim to produce high-quality clinical trial data while safeguarding participant rights and data integrity.

A Data Safety Monitoring Board (DSMB), whose members are independent of the trial investigators and free of competing interests, will be established to oversee participant safety throughout the study. The board will review unblinded safety data, including adverse events, laboratory abnormalities, and study discontinuations, and based on its assessments may recommend continuation, modification, or early termination of the trial. All recommendations will be formally documented and communicated to the principal investigator and the institutional review board.

### Sample size calculation

Pilot studies are an essential first step to assess the practicality and feasibility of methods and procedures intended for larger-scale investigation (20–22). They should not be used to estimate effect sizes, to provide power calculations for statistical tests or to perform exploratory analyses of efficacy (20). The present work is a pilot randomized controlled trial (RCT) primarily designed to evaluate the feasibility, safety, and preliminary signal of QHF on the incidence of END in AIS patients. No formal sample size calculation was performed; the target of 36 participants per group was considered adequate for exploring the feasibility of study procedures.

### Statistical analyses

Statistical analyses will be conducted using three datasets: the full analysis set (FAS), the per-protocol set (PPS), and the safety set (SS). The FAS will include all randomized participants with at least one post-baseline assessment, with missing data imputed using the multiple-imputation method (23). Sensitivity analyses will be conducted using last observation carried forward (LOCF), worst-case, and best-case scenarios to assess the robustness of the primary outcome under different missing data assumptions. The PPS will comprise only those who fully comply with the protocol without major deviations. The SS will include all participants who receive at least one dose of the study drug and undergo at least one safety evaluation. Both efficacy and safety analyses will follow the intention-to-treat principle. Qualitative variables will be summarized using frequencies and percentages, while quantitative variables will be presented as means ± standard deviations for normally distributed data or medians with interquartile ranges for non-normally distributed data.

For the primary efficacy outcome, the incidence of END will be compared between groups using the chi-square or Fisher exact test, as appropriate. Risk differences and 95% confidence intervals will be reported. Secondary efficacy outcomes such as BI scores, changes in NIHSS scores, and relevant biomarkers will also be compared between groups using Student's *t*-test or the Wilcoxon rank-sum test, with mean differences and 95% confidence intervals reported where applicable. Additionally, the proportion of patients with a mRS grade ≤ 2 and the incidence of AEs will be compared using the chi-square test or Fisher exact test, as appropriate.

Prespecified subgroup analyses for the primary outcome will be performed according to the following baseline characteristics: age (>65 years vs. ≤ 65 years); sex (female vs. male); symptom onset to randomization time ( ≤ 24 h vs. >24 h); medical history (hypertension, diabetes mellitus, coronary heart disease, stroke, and hypercholesterolemia); smoking history; Trial of Org 10172 in acute stroke treatment (TOAST) classification; presence of large artery stenosis; stroke severity based on baseline NIHSS score ( ≤ 5 vs. >5); and severity of phlegm-heat syndrome.

No formal interim analysis is planned for this trial. However, an independent Data Safety Monitoring Board will regularly review safety data throughout the study to ensure participant safety. The DSMB may recommend early termination if unexpected safety concerns arise.

All statistics will be evaluated using two-sided tests, and *p* < 0.05 will be considered statistically significant. Statistical analysis will be performed using SAS version 9.4 (SAS Institute Inc.).

## Discussion

In recent years, END in AIS has attracted increasing attention. Randomized controlled trials of argatroban and tirofiban for END have yielded encouraging results, igniting fresh hope for tackling this disorder (24, 25). Nevertheless, some clinical challenges should not be underestimated. First, the pathogenesis of END in AIS is multifactorial, most commonly attributable to failure of collateral circulation with secondary hypoperfusion of the ischemic penumbra, as well as to clot progression, recurrent stroke, cytotoxic cerebral edema, hemorrhagic transformation, re-occlusion of a recanalized artery, and seizures (8). These mechanisms interact to cause rapid neurological deterioration, significantly increasing the risks of long-term disability and death. Second, the early identification of patients at high risk of END is still problematic. Although numerous prediction models for END in AIS have been proposed, none has gained widespread acceptance or entered routine clinical use, primarily owing to inconsistent END definitions, small sample sizes, lack of external validation, high patient heterogeneity, and operational complexity (4, 26). In addition, current reperfusion, antithrombotic, and neuroprotective interventions have shown limited efficacy against END (9). Consequently, the development of safe and effective pharmacotherapies specifically targeting END in AIS is a critical unmet clinical need.

Rooted in thousands of years of continuous development, TCM has accumulated systematic experience in preventing and treating AIS, and it has fostered a number of well-established drugs with verified efficacy. During the past decade, research in this area has accelerated, generating a succession of high-quality clinical evidence that supplies new therapeutic options for this condition (10–12). Our earlier research highlighted the phlegm-heat syndrome as a key pattern in AIS (13). Timely heat-clearing and phlegm-resolving therapy is essential for symptom resolution, neurological recovery, and END prevention (14, 27). Accordingly, we developed the Qingre Huatan Formula (QHF), comprising Gastrodiae Rhizoma, Salviae Miltiorrhizae Radix et Rhizoma, Trichosanthis Fructus, Arisaema cum Bile, Rhei Radix et Rhizoma, Gardeniae Fructus and Acori Tatarinowii Rhizoma. According to traditional Chinese medicine theory, these herbs act synergistically to extinguish wind-phlegm, clear heat and purge the bowels, and open the orifices to unblock collateral circulation.

This study has several strengths in the field of preventing END in AIS. First, we pioneered the use of a RCT design to systematically evaluate the efficacy and safety of traditional Chinese medicine interventions for END. Second, by enrolling only confirmed high-risk END patients, we enhanced the precision of the estimates and statistical power.

We acknowledge that the optimal definition of END remains debated, with no universally accepted threshold, as highlighted in a systematic review (4). While some studies have used a ≥4-point NIHSS increase as the threshold for clinically meaningful deterioration, recent randomized trials published in JAMA Neurology (25, 28) have successfully employed the ≥ 2-point definition as a primary or key secondary endpoint, demonstrating its ability to capture clinically significant treatment effects. Evidence from cohort studies further suggests that a ≥2-point increase is a sensitive predictor of poor outcomes and in-hospital mortality, and this threshold may be particularly appropriate for identifying early deterioration in high-risk populations where timely intervention is critical (29, 30). Future larger trials may consider sensitivity analyses using different END thresholds to further inform this methodological question.

Nevertheless, certain limitations should not be ignored. First, this is a single-center pilot trial with a relatively small sample size, limiting statistical power. Second, as an exploratory study, it has not yet completed long-term follow-up or multi-center validation, and thus cannot provide high-level evidence for subsequent large-scale trials.

In summary, the QUIET trial aims to determine whether the early initiation of QHF within 48 h of stroke onset reduces the incidence of END in AIS patients compared with placebo. If the results are positive, this study will provide robust evidence for the prevention and treatment of END in AIS, thereby reshaping and optimizing therapeutic strategies for this condition.
