# Supplementary material for: Efficacy and safety of Qingre Huatan Formula for the prevention of early neurological deterioration in patients with acute ischemic stroke (QUIET): rationale and design for a randomized double-blind placebo-controlled study
Source: Front Med (Lausanne). 2026 Apr 13;13:1754648. doi: 10.3389/fmed.2026.1754648 (PMC13111062; doi:10.3389/fmed.2026.1754648)
Supplement: Supplementary file 1 [file Table_1.docx]

**Supplementary materials**

**The composition of Qingre Huatan Formula granules**

| English Name | Latin Name | Chinese Pinyin Name | Amount (g) |
| --- | --- | --- | --- |
| Tall Gastrodia Tuber | Gastrodiae Rhizoma | Tianma | 10 |
| Dan-Shen Root | Salviae Miltiorrhizae Radix et Rhizoma | Danshen | 20 |
| Snakegourd Fruit | Trichosanthis Fructus | Gualou | 15 |
| Bile Arisaema | Arisaema Cum Bile | Dannanxing | 6 |
| Rhubarb | Rhei Radix et Rhizoma | Dahuang | 6 |
| Cape Jasmine Fruit | Gardeniae Fructus | Zhizi | 6 |
| Grassleaf Sweetflag Rhizome | Acori Tatarinowii Rhizoma | Shichangpu | 12 |

Table 1 The composition of Qingre Huatan Formula granules.

**Fingerprint of QHF**

【Determination by High Performance Liquid Chromatography (HPLC) (General Rule 0512, Chinese Pharmacopoeia 2020 Edition)】

****Method:****

****1 Chromatographic Conditions and System Suitability Test****
Use octadecylsilane chemically bonded silica as the stationary phase (Chromatographic column: Welch Ultimate AQ-C18, 250 mm × 4.6 mm, 5 μm); use acetonitrile as mobile phase A and 0.1% phosphoric acid solution as mobile phase B, following the gradient elution program specified in the table below; the detection wavelength is 220 nm; the flow rate is 1.0 ml per minute; the column temperature is 30°C. The number of theoretical plates calculated based on the geniposide peak should be not less than 3000.

Table 2 Gradient elution program.

| Time (minutes) | Mobile phase A (Acetonitrile, %) | Mobile phase B (0.1% phosphoric acid solution, %) |
| --- | --- | --- |
| 0～20 | 5→19 | 95→81 |
| 20～40 | 19→32 | 81→68 |
| 40～70 | 32→50 | 68→50 |
| 70～85 | 50→90 | 50→10 |
| 85～86 | 90→5 | 10→95 |
| 86～95 | 5 | 95 |

**2 Preparation of Reference Substance Solution**

Accurately weigh an appropriate amount of geniposide reference substance, dissolve in methanol, and prepare a solution containing 0.2 mg per 1 ml.

**3 Preparation of Test Solution**

Take an appropriate amount of this product, grind to fine powder. Accurately weigh about 0.5 g, place in a stoppered conical flask. Accurately add 25 ml of methanol, weigh, and treat with ultrasonication for 30 minutes (power 500 W, frequency 40 kHz). Allow to cool, weigh again, replenish the lost weight with methanol, and mix well. Filter through a 0.45 μm microporous membrane and use the subsequent filtrate as the test solution.

**4 Procedure**

Accurately draw 10 μl each of the reference substance solution and the test solution, inject into the liquid chromatograph, determine, and record the chromatograms.

**5 Result Determination**

The fingerprint of the test solution should show a peak (Peak S) with the same retention time as the reference substance chromatographic peak. The chromatogram of the test solution should be essentially consistent with the reference fingerprint, showing the corresponding 18 common peaks. Calculated using the “Chromatographic Fingerprint Similarity Evaluation Software System for Traditional Chinese Medicine (2012 Edition)”, the similarity between the fingerprint of the test solution and the reference fingerprint should be not less than 0.90.

**Results:**

**1 Identification of Common Peaks**

Four batches of NYSPZ002 granules were determined using the method specified in the [Fingerprint] section of this product’s quality standard draft. The obtained fingerprints were analyzed. Peaks with good stability and appropriate response values in the fingerprints of the four batches were selected as common peaks. A total of 18 common peaks were identified. The results are shown in Fig. 1.


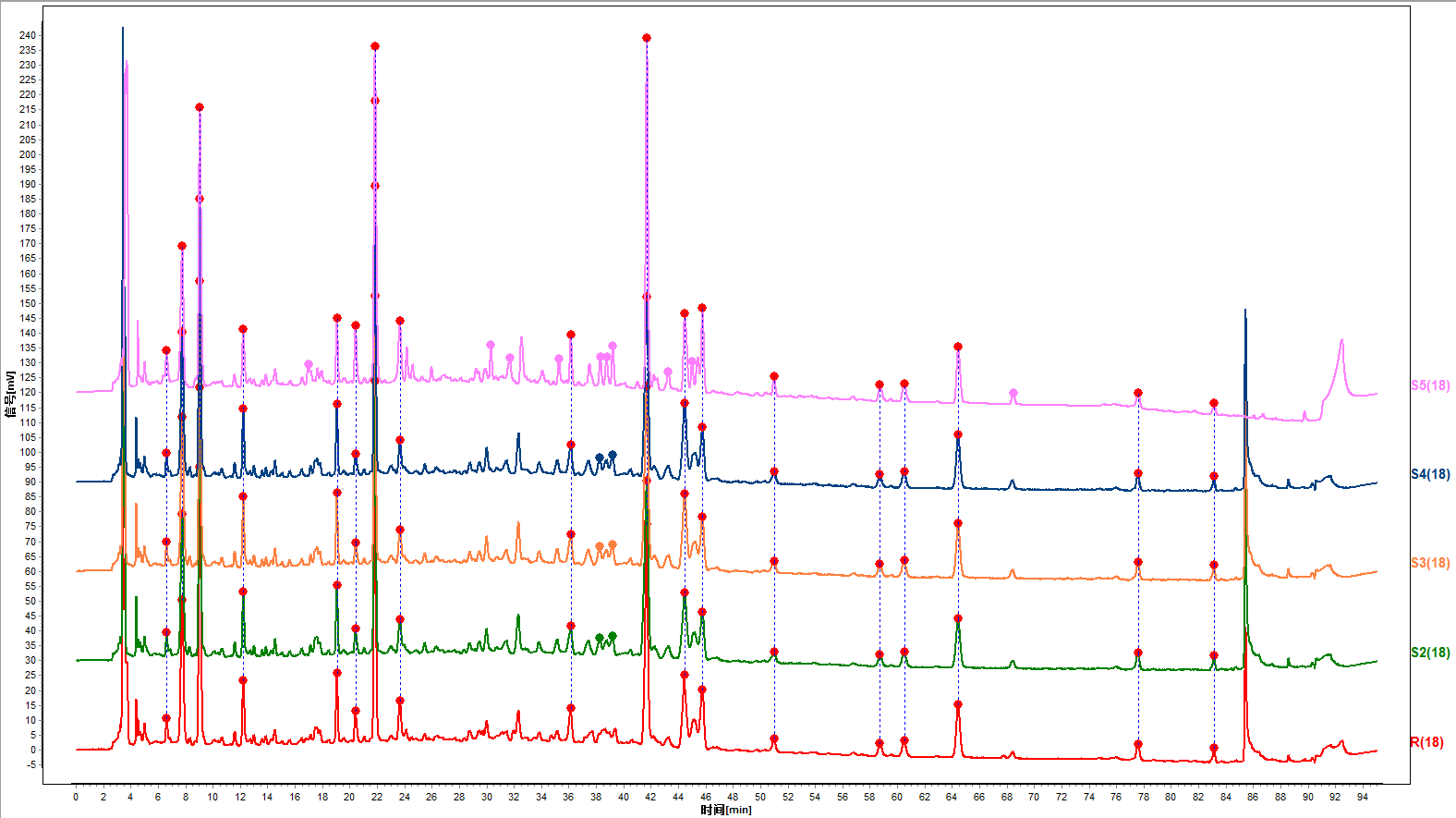


Figure 1 Common Pattern of Fingerprints of 4 Batches of NYSPZ002 Granules (1) S2. NYSPZ002 Granules (Batch No.: 241101); (2) S3. NYSPZ002 Granules (Batch No.: 241102); (3) S4. NYSPZ002 Granules (Batch No.: 241103); (4) S5. NYSPZ002 Granules (Batch No.: 240401)

**2 Establishment of Reference Fingerprint**

Using the “Chromatographic Fingerprint Similarity Evaluation Software System for Traditional Chinese Medicine (2012 Edition)” promulgated by the Chinese Pharmacopoeia Commission, the chromatographic peaks of the fingerprints of the four batches of NYSPZ002 granules were automatically matched to form a common pattern, and a reference fingerprint was established. The results are shown in Fig. 2.


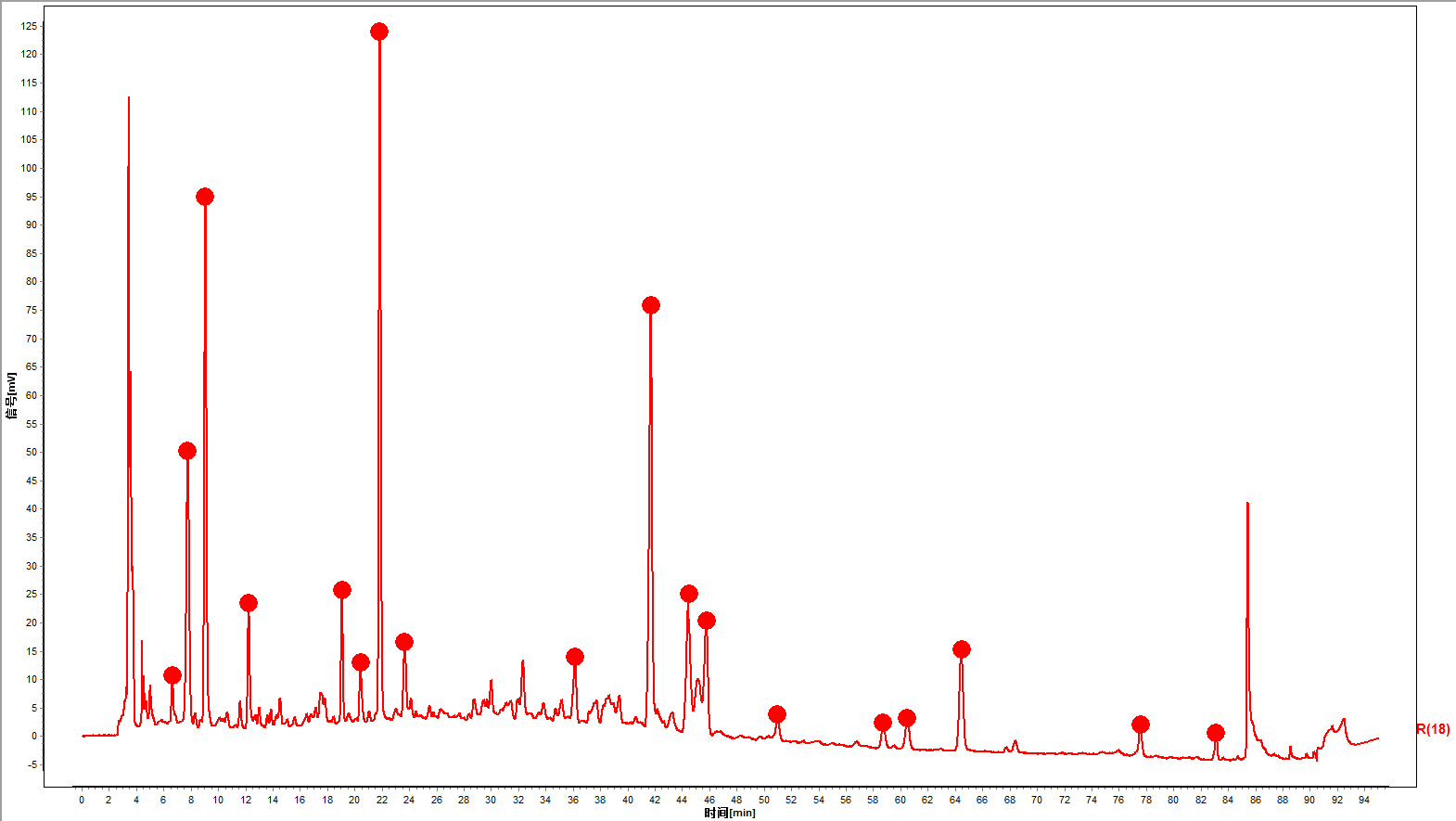


Figure 2 Reference Fingerprint. Among the 18 common peaks: Peak 7 (S): Geniposide; Peak 10: Salvianolic acid B; Peak 15: Aloe-emodin; Peak 16: Rhein; Peak 17: Emodin; Peak 18: Chrysophanol

**3 Similarity Calculation Results and Limit Determination**

The fingerprint of the test solution should show a peak with the same retention time as the reference substance chromatographic peak. The chromatogram of the test solution should be essentially consistent with the reference fingerprint, showing the corresponding 18 common peaks. Using the TCM fingerprint similarity evaluation system, the similarity was calculated based on the common peaks. The results are shown in Figure 3.4.3-7. The similarities between the fingerprints of the test solutions and the reference fingerprint were all greater than 0.90. It is tentatively specified that the similarity limit should be not less than 0.90.

Table 3 Similarity evaluation results of 4 batches of NYSPZ002 granules.

|  | R | S2 | S3 | S4 | S5 |
| --- | --- | --- | --- | --- | --- |
| R | 1.000 | 1.000 | 0.999 | 0.999 | 0.993 |
| S2 | 1.000 | 1.000 | 0.999 | 0.999 | 0.990 |
| S3 | 0.999 | 0.999 | 1.000 | 1.000 | 0.986 |
| S4 | 0.999 | 0.999 | 1.000 | 1.000 | 0.986 |
| S5 | 0.993 | 0.990 | 0.986 | 0.986 | 1.000 |
| Calibration Peak Group | 0 |  |  |  |  |

**Ischemic Stroke TCM Syndrome Diagnostic Scale (ISDS): Detailed Scoring Rules**

**I. Scoring Procedure**

1. Data Collection by Four Diagnostic Methods

Trained physicians shall collect clinical information through inspection, listening and smelling, inquiry, and palpation. For each item listed in the scale, the physician shall check the corresponding box “□” if the symptom or sign is present.

2. Item Scoring

Each checked item receives its assigned score. The items and scores for each syndrome element are as follows:

| **Syndrome Element** | **Items** | **Total Score** |
| --- | --- | --- |
| **1 Internal Wind** | □ Acute onset within the last 48 hours (10 points)  □ Aggravation or fluctuation of the condition within the last 48 hours (10 points)  □ Dizziness and vertigo (10 points)  □ Deviation of the eye (10 points)  □ Tremors in the hands, feet, or jaw (10 points)  □ Limb rigidity (10 points)  □ Limb spasm/cramp (10 points)  □ Convulsions (10 points)  □ Shortened/contracted tongue (10 points)  □ Trembling tongue (10 points) |  |
| **2 Internal Fire** | **□** Flushed face (4 points)  □ Malar flush (2 points)  □ Red eyes (7 points)  □ Dry mouth (3 points)  □ Thirst with preference for cold drinks (4 points)  □ Dry tongue (2 points)  □ Dry and cracked lips (6 points)  □ Bitter taste in the mouth (2 points)  □ Foul breath (4 points)  □ Yellow sputum (1 point)  □ Dysphoria (1 point)  □ Agitation and restlessness (3 points)  □ Acid regurgitation (2 points)  □ Dark urine (4 points)  □ Dry stools (1 point)  □ Red or crimson tongue (2 points)  □ Yellow tongue coating (10 points)  □ Dry tongue coating (2 points)  □ Rapid pulse (5 points)  □ Wiry pulse (2 points)  □ Slippery pulse (1 point) |  |
| **3 Phlegm-Dampness** | **□** Blank expression or taciturnity (1 point)  □ Dull/Stuporous expression (2 points)  □ Obesity (1 point)  □ Distending pain in the head (6 points)  □ Heavy sensation in the head (3 points)  □ Dizziness or light-headedness (1 point)  □ Sticky sensation in the mouth (2 points)  □ Thirst with no desire to drink (2 points)  □ Expectoration or wheezing with sputum (10 points)  □ Poor appetite (2 points)  □ Loose stools (4 points)  □ Enlarged tongue (3 points)  □ Teeth-marked tongue (2 points)  □ Thick tongue coating (3 points)  □ Greasy tongue coating (8 points)  □ Slippery tongue coating (9 points)  □ Slippery pulse (6 points) |  |
| **4 Blood Stasis** | □ Dark or blackish complexion (9 points)  □ Purplish or dark red lips (8 points)  □ Rough skin (4 points)  □ Fixed pain (5 points)  □ Purple or dark tongue (10 points)  □ Petechiae or ecchymosis on the tongue (10 points)  □ Purplish sublingual veins (10 points)  □ Varicose sublingual veins (8 points)  □ Rough/choppy pulse (8 points)  □ Knotted or intermittent pulse (1 point) |  |
| **5 Qi Deficiency** | **□** Listlessness (2 points)  □ Fatigue (5 points)  □ Pale white complexion (9 points)  □ Sallow complexion (3 points)  □ Pale lips (9 points)  □ Shortness of breath (8 points)  □ Weak voice (9 points)  □ Edema of hands or feet (3 points)  □ Spontaneous sweating (2 points)  □ Stool initially hard then loose (3 points)  □ Fecal or urinary incontinence (5 points)  □ Pale tongue (3 points)  □ Enlarged or teeth-marked tongue (1 point)  □ Moderate/Slow pulse (5 points)  □ Thready pulse (5 points)  □ Deep pulse (4 points)  □ Weak pulse (4 points)  □ Knotted or intermittent pulse (1 point) |  |
| **6 Yin Deficiency** | □ Malar flush (10 points)  □ Dry tongue (5 points)  □ Heat in the palms and soles (5 points)  □ Feverish sensation in the chest, palms, and soles (10 points)  □ Night sweats (10 points)  □ Crimson tongue (10 points)  □ Thin and wasted tongue body (10 points)  □ Peeled/Exfoliated tongue coating (10 points)  □ Bright red tongue without coating (10 points)  □ Thready, wiry, or rapid pulse (5 points) |  |

3. Calculating the Total Score for Each Syndrome Element

Sum the scores of all checked items under each syndrome element to obtain the total score for that element.

**II. Diagnostic Criteria**

1. Diagnostic Threshold for Syndrome Elements

According to the methodological studies and clinical validation of the scale, the unified diagnostic threshold for each syndrome element is: Total Score ≥ 10 points.

2. Diagnosis of a Single Syndrome Element

A syndrome element is diagnosed when its total score reaches or exceeds 10 points.

3. Diagnosis of Combined Syndromes

For the diagnosis of combined syndromes (e.g., Phlegm-Heat Syndrome in this study), multiple related syndrome elements must meet the diagnostic criteria simultaneously. For example: Diagnostic criteria for Phlegm-Heat Syndrome: total score for “Phlegm-Dampness” ≥ 10 points and total score for “Internal Fire” ≥ 10 points.

**III. Scoring Instructions**

1. Source of Scores: The item scores in this scale were derived through a two-round Delphi expert consultation and clinical data collection from 14,880 patient visits, with weights assigned using Logistic discriminant analysis.

2. Target Population: This scale is applicable for syndrome diagnosis in patients with acute ischemic stroke and those in the early recovery phase.

3. Requirements for Raters: It is recommended that the assessment be performed by physicians with the title of attending physician or above who have received standardized training on this scale.

4. Consistency Test: The Kappa statistic can be used to test inter-rater reliability. A Kappa value ≥ 0.80 indicates good consistency.
