# Supplementary material for: Efficacy and safety of Qingre Huatan Formula for the prevention of early neurological deterioration in patients with acute ischemic stroke (QUIET): rationale and design for a randomized double-blind placebo-controlled study
Source: Front Med (Lausanne). 2026 Apr 13;13:1754648. doi: 10.3389/fmed.2026.1754648 (PMC13111062; doi:10.3389/fmed.2026.1754648)
Supplement: Supplementary file 2 [file Table_2.docx]

**Supplemental Table 1: SPIRIT 2025 expanded checklist detailing items to address in randomized trial protocols, based on Explanation and Elaboration document**

| **SPIRIT 2025** | | | **Expanded items to report** |  |
| --- | --- | --- | --- | --- |
| **Section / Topic** | **No** | **SPIRIT 2025 checklist item description** |  | **Page** |
| **Administrative information** | | |  |  |
| Title and structured summary | 1a | Title stating the trial design,  population, and interventions, with identification as a protocol | • Descriptive title stating:  o Trial design (e.g., parallel group randomised trial)  o Conceptual framework (e.g., superiority, non-inferiority)  o Trial phase (if applicable)  o Population  o Intervention/comparator  o Objective or primary outcome  o “Protocol” | 1 |
|  | 1b | Structured summary of trial  design and methods, including items from the World Health  Organization Trial Registration Data Set | • Relevant items from the WHO Trial Registration Data Set:  o Primary Registry and Trial Identifying Number  o Secondary Identifying Numbers  o Source(s) of Monetary or Material Support  o Primary Sponsor  o Contact for Public Queries  o Contact for Scientific Queries  o Public Title  o Scientific title  o Countries of Recruitment  o Health Condition(s) or Problem(s) Studied  o Intervention(s)  o Key Inclusion and Exclusion Criteria  o Study Type  o Date of First Enrollment (planned)  o Sample Size  o Primary outcome(s)  o Key Secondary outcome(s)  o Ethics Review | 1-2 |

|  |  |  | o Individual Trial Participant Data sharing statement |  |
| --- | --- | --- | --- | --- |
| Protocol version | 2 | Version date and identifier | • Version date  • Version identifier (e.g., Version 2.0)  • List of changes made relative to the previous protocol version, with reasons | 4 |
| Roles and responsibilities | 3a | Names, affiliations, and roles of protocol contributors | For each protocol contributor:  • Name  • Affiliation  • Description of contributions, including use of artificial intelligence technologies, if applicable | 1, 11-12 |
|  | 3b | Name and contact information for the trial sponsor | For the trial sponsor (e.g. individual, company, institution, or organization):  • Name  • Contact information  • Regulatory agency identifying number (if applicable) | 12 |
|  | 3c | Role of trial sponsor and  funders in design, conduct,  analysis, and reporting of trial; including any authority over  these activities | For the Sponsor and funders:  • Roles and responsibilities in trial design, conduct, data analysis and interpretation, manuscript writing, and results dissemination  • Who will make final decision regarding the above trial aspects  • Whether the sponsor or funder will have the right to review or comment on the trial manuscript  • Any mechanisms used to mitigate funder influence. If the funder will have no direct involvement in the trial, then this should be explicitly stated. | 12 |
|  | 3d | Composition, roles, and  responsibilities of the  coordinating site, steering  committee, endpoint  adjudication committee, data management team, and other individuals or groups  overseeing the trial, if applicable | For each trial committee:  • Roles and responsibilities  • Relationship to trial sponsor and funders  • Outline of membership (e.g., clinician, biostatistician, patient)  • Names of Chairs and members, when known  Examples of committees include:  • Trial Steering Committee (executive decisions)  • Trial Management Group (day-to-day trial conduct)  • Data Monitoring Committee (review of accumulating data)  • Endpoint adjudication committee  • Data management team  • Other individuals or groups overseeing the trial | 8-9 |

| **Open science** | | |  |  |
| --- | --- | --- | --- | --- |
| Trial registration | 4 | Name of trial registry,  identifying number (with URL), and date of registration. If not yet registered, name of  intended registry | • Name of registry  • Trial registry identifying number  • URL to registry record  • Date of registration | 3-4 |
| Protocol and statistical analysis plan | 5 | Where the trial protocol and statistical analysis plan can be accessed | • Where the protocol will be accessible (e.g., publication, repository such as Open Science Framework, trial registry)  • Where the full statistical analysis plan will be accessible | 9-10 |
| Data sharing | 6 | Where and how the individual de-identified participant data (including data dictionary),  statistical code, and any other materials will be accessible | • What data and materials will be shared; for example:  o De-identified participant data, data dictionary, analytical code used to process the data  o Materials associated with the intervention (e.g., handbook or video for non- pharmacological interventions)  • How the data and materials will be shared with trial investigators and external parties, including:  o Application process to access the data (if applicable)  o Data transfer process (e.g., via repository or direct transfer to user)  o Any plans to obtain consent from participants  • If no sharing is planned, this should be clearly stated with an explanation | 8-9 |
| Funding and conflicts of interest | 7a | Sources of funding and other support (e.g., supply of drugs) | For each funding source:  • Name of funder  • Type of funding:  o Direct monetary support  o Indirect support (free trial drugs, equipment, or services such as statistical analysis or use of medical writers) | 12 |
|  | 7b | Financial and other conflicts of interest for principal  investigators and steering committee members | • Conflicts of interests for principal trial investigators and members of key committees involved in the trial (e.g. steering and data monitoring committees), including any of the following support received:  o Financial: Salary support or grants; ownership of stock or options; honoraria (e.g., for advice, authorship, or public speaking); paid consultancy or service on advisory boards; and holders of patents or patents pending  o Non-financial: academic commitments; personal or professional  relationships; and other affiliations with special interests or advocacy positions | 12 |

|  |  |  | • Any procedures planned to reduce the potential influence of conflicts of interest on the trial’s design, conduct, analysis, or reporting  • If no conflicts of interest, this should be clearly stated |  |
| --- | --- | --- | --- | --- |
| Dissemination policy | 8 | Plans to communicate trial  results to participants,  healthcare professionals, the public, and other relevant  groups (e.g., reporting in trial registry, plain language  summary, publication) | • Plan to disseminate trial results to participants, healthcare professionals, the public, and other relevant groups (e.g., reporting results in trial registry, preprint, plain  language summary, publication in open access journal)  • Process and timeframe for approving and submitting reports for dissemination  • Authorship guidelines | 12 |
| **Introduction** | | |  |  |
| Background and rationale | 9a | Scientific background and  rationale, including summary of relevant studies (published and unpublished) examining  benefits and harms for each intervention | • Importance of the research question  • Why a new trial is needed in the context of available evidence  o Explanation of how the intervention might work  o Pre-trial evidence of the benefits and harms of the intervention  o Reference to systematic review(s) of relevant trials; if none available, a summary of relevant evidence based on a systematic search | 3 |
|  | 9b | Explanation for choice of comparator | • Why the particular comparator group was chosen  • Whether the comparator represents standard of care | 3 |
| Objectives | 10 | Specific objectives related to benefits and harms | • Trial objectives related to benefits and harms including:  o Participants  o Intervention  o Comparator  o Primary outcome(s)  o Time point of main interest  • Description of trial estimand(s), as appropriate | 3 |
| **Methods: Patient and public involvement, trial design** | | |  |  |
| Patient and public involvement | 11 | Details of, or plans for, patient or public involvement in the  design, conduct, and reporting of the trial | • Planned methods of patients and public involvement at different trial stages (e.g., design, conduct, reporting)  • Who is involved (e.g., patients, carers, members of the public)  • If no patient or public involvement planned, this should be stated | 4-5 and Table 1 |
| Trial design | 12 | Description of trial design  including type of trial (e.g.,  parallel group, crossover),  allocation ratio, and framework | • Type of trial design (e.g., parallel group)  • Conceptual framework (e.g.,superiority, non-inferiority, or equivalence)  • Unit of randomisation (e.g., individual participant)  • Allocation ratio (e.g., 1:1) | 3-5 |

|  |  | (e.g., superiority, equivalence, non-inferiority, exploratory) |  |  |
| --- | --- | --- | --- | --- |
| **Methods: Participants, interventions, and outcomes** | | |  |  |
| Trial setting | 13 | Settings (e.g., community,  hospital) and locations (e.g.,  countries, sites) where the trial will be conducted | • Setting of participant recruitment (e.g., primary or tertiary care; outpatient community or hospital clinic; inpatient unit)  • Location(s) where the trial will be carried out (e.g., country, city)  • Planned number of sites | 4 |
| Eligibility criteria | 14a | Eligibility criteria for participants | • Specific inclusion and exclusion criteria defining the trial population to be randomised | Table 1 |
|  | 14b | If applicable, eligibility criteria  for sites and for individuals who will deliver the interventions  (e.g., surgeons,  physiotherapists) | If applicable:  • Eligibility criteria for sites (e.g., site volume for surgical procedure)  • Eligibility criteria for individuals delivering the interventions (e.g., surgeons,  physiotherapists), such as professional qualifications, years in practice, skills, or validation of specific training before trial initiation | 4 and 8 |
| Intervention and comparator | 15a | Intervention and comparator  with sufficient details to allow replication including how,  when, and by whom they will  be administered. If relevant,  where additional materials  describing the intervention and comparator (e.g., intervention manual) can be accessed | • Details of each intervention and comparator to allow replication, including:  o Components of the intervention and comparator  o How they will be administered  o When and for how long they will be administered  o Any procedure for tailoring the intervention to individual participants  o Any physical or informational materials to be used as part of the  intervention/comparator (e.g., instruction manual) and where the materials will be made accessible  • When comparator group is “usual care”:  o Description of usual care and any plans to track and measure it during the trial  o Whether the intervention group will also receive usual care | 6 |
|  | 15b | Criteria for discontinuing or  modifying allocated  intervention/comparator for a trial participant (e.g., drug dose change in response to harms,  participant request, or  improving/worsening disease) | • Criteria to guide modifications to trial intervention/comparator (e.g., drug dose  change in response to harms, participant request, or improving/worsening disease)  • Criteria to guide discontinuation of trial intervention/comparator | 6 |
|  | 15c | Strategies to improve adherence to | • Strategies for improving fidelity of care providers and adherence of participants to intervention/comparator protocols, if applicable | 6 |

|  |  | intervention/comparator  protocols, if applicable, and any procedures for monitoring  adherence (e.g., drug tablet return, sessions attended) | • When and how fidelity of care providers and adherence of participants to intervention/comparator protocols will be assessed, if applicable  • Where appropriate, prespecified definition for classifying participants as being treated as planned or not |  |
| --- | --- | --- | --- | --- |
|  | 15d | Concomitant care that is  permitted or prohibited during the trial | • Relevant concomitant care that is allowed (e.g., rescue interventions) or prohibited during the trial  • Any plans to record concomitant care received, including “usual care” | 6 |
| Outcomes | 16 | Primary and secondary  outcomes, including the specific measurement variable (e.g.,  systolic blood pressure),  analysis metric (e.g., change  from baseline, final value, time to event), method of  aggregation (e.g., median,  proportion), and time point for each outcome | • Specification of which outcomes are primary and secondary  • Rationale for the choice of trial outcomes and whether they are part of a core outcome set  • For each outcome:  o Specific variable to be measured (e.g., Beck Depression Inventory score, all- cause mortality), with definition where relevant  o Analysis metric for each participant (e.g., change from baseline, end value, time-to-event)  o Summary measure for each study group (e.g., mean, proportion with score > 2)  o Time point of interest for analysis (e.g., 3 months) | 6-7 |
| Harms | 17 | How harms are defined and will be assessed (e.g.,  systematically, non-  systematically) | For each systematically assessed harm (active/targeted surveillance):  • Definition and measurement (e.g., name of validated questionnaire)  • Where appropriate, the metrics, method of aggregation, and time point of interest for analysis  • Procedures for harms assessment, including:  Who will do the assessment, and whether they will be blinded to the allocated trial group  o Assessment time points and overall time period for recording harms  For each non-systematically assessed harm (passive surveillance):  • How data will be collected  • Assessment time points and overall time period for recording harms  • Process for coding each adverse event and grading its severity, including:  o Who will do the coding and severity grading, and whether they will be blinded to the allocated trial group  o Which coding and severity grading systems will be used, if any | 7 |

|  |  |  | For grouping of harms by seriousness, severity, body system, discontinuation of intervention (due to harms), and causality:  • Definitions of grouping categories  • Who will do the grouping, and whether they will be blinded to the allocated trial group  If relevant:  • Process of reporting important adverse events to applicable groups (e.g, sponsor, regulator, data monitoring committee) |  |
| --- | --- | --- | --- | --- |
| Participant timeline | 18 | Time schedule of enrolment,  interventions (including any  run-ins and washouts),  assessments, and visits for  participants. A schematic  diagram is highly recommended (see Figure). | • Schematic diagram outlining the schedule and time commitment for trial participants, including:  o Timeline of trial visits starting from eligibility screening to trial close-out  o Timeline of interventions including any run-in and washout periods  o Procedures and assessments performed at each visit, referencing specific data collection forms, if relevant | Table 2 |
| Sample size | 19 | How sample size was  determined, including all  assumptions supporting the sample size calculation | For sample size calculations:  • Primary outcome (and any other outcome) on which the calculations are based  • Outcome values (e.g., proportion) assumed for each group, with rationale  • Target difference in outcome values between trial groups (including common standard deviation for continuous outcomes), with rationale  • Statistical significance level or α (type I) error  • Statistical power or β (type II) error  • Any upward adjustments (e.g., accounting for missing data or non-adherence)  • Target sample size per trial group  • Any software used | 9 |
| Recruitment | 20 | Strategies for achieving  adequate participant enrolment to reach target sample size | • Planned strategies to promote adequate enrolment (e.g., advertisements, pre- screening of health records, reducing participant burden)  • Where participants will be recruited (e.g., primary care clinic, community), by whom (e.g., surgeon), and when (e.g., time period after diagnosis) | 4 |
| **Methods: Assignment of interventions** | | |  |  |
| Randomization: |  |  |  |  |

| Sequence generation | 21a | Who will generate the random allocation sequence and the  method used | • Who will generate the allocation sequence  • Method of sequence generation (e.g., computerized random number generator)  • Any software used | 5 |
| --- | --- | --- | --- | --- |
|  | 21b | Type of randomization (simple or restricted) and details of any factors for stratification. To  reduce predictability of a  random sequence, other details of any planned restriction (e.g., blocking) should be provided in a separate document that is  unavailable to those who enroll participants or assign  interventions | • Type of randomization: simple versus restricted (e.g., blocked); fixed versus adaptive (e.g., minimization); and where relevant, the reasons for such choices  • If applicable, factors (e.g., trial site, sex, disease stage) to be used for stratification, including categories and relevant cut-off boundaries.  • For restricted randomization: aside from the above, all other details on restriction (including minimization) should be provided in a separate document in order to  reduce predictability of the random sequence | 5 |
| Allocation concealment mechanism | 22 | Mechanism used to implement the random allocation  sequence (e.g., central  computer/telephone;  sequentially numbered,  opaque, sealed containers),  describing any steps to conceal the sequence until  interventions are assigned | • How the individuals enrolling participants will be kept unaware of the next trial  group assignment in the random sequence (not to be confused with blinding) | 5 |
| Implementation | 23 | Whether the personnel who will enroll and those who will assign participants to the  interventions will have access to the random allocation  sequence | • Who will have access to the random allocation sequence  • Who will enroll participants  • Who will assign participants to interventions  • Whether the personnel enrolling and assigning participants will have no access to the random allocation sequence  When individuals involved in sequence generation and allocation concealment are the same individuals involved in the implementation of assignment:  • How and where the random allocation list will be securely stored  • Any mechanisms to prevent those enrolling and assigning participants from accessing the list | 5 |
| Blinding | 24a | Who will be blinded after  assignment to interventions | • Who will be blinded to treatment assignments:  o Trial participants | 5 |

|  |  | (e.g., participants, care  providers, outcome assessors, data analysts) | o Care providers (i.e., those administering the intervention)  o Outcome assessors (i.e., those who determine if a participant experienced the outcome of interest). e.g., the participant (for patient-reported  outcomes), care provider, or independent observer  o Data analysts performing the statistical analysis |  |
| --- | --- | --- | --- | --- |
|  | 24b | If blinded, how blinding will be achieved and description of the similarity of interventions | For blinded trials:  • Mechanism to establish blinding (e.g., identical placebo, double-dummy)  • Any similarities or differences in characteristics (e.g. appearance, taste) of the interventions being compared  • Any procedures intended to maintain blinding and reduce risk of accidental unblinding  • Any procedures intended to evaluate blinding procedures (e.g., pre-trial testing of blinding procedures) | 5 |
|  | 24c | If blinded, circumstances under which unblinding is permissible, and procedure for revealing a  participant’s allocated  intervention during the trial | For blinded trials:  • Circumstances under which unblinding is permissible during the trial (e.g., to reduce immediate risk for a participant)  • Procedure for revealing a participant’s allocated intervention during the trial | 5 |
| **Methods: Data collection, management, and analysis** | | |  |  |
| Data collection methods | 25a | Plans for assessment and  collection of trial data, including any related processes to  promote data quality (e.g.,  duplicate measurements,  training of assessors) and a  description of trial instruments (e.g., questionnaires, laboratory tests) along with their reliability and validity, if known.  Reference to where data  collection forms can be  accessed, if not in the protocol | • Who will assess the outcome (e.g., participant, doctor, nurse, caregiver)  • Who will collect the data (e.g., participant, doctor, nurse, caregiver)  • Mode of data collection (e.g., paper-based data collection, mobile devices)  • Description of data collection instruments (e.g. validated questionnaires, laboratory instruments), including reliability and validity  • Processes to promote quality of data collection (e.g., duplicate measurements, training of assessors)  • Where the data collection form can be accessed (e.g., appendix, link to repository)  • Any pilot testing and assessment of reliability and validity of the forms, if performed | 8-9 |
|  | 25b | Plans to promote participant  retention and complete follow- up, including list of any | • Retention strategies to promote complete follow-up and prevent missing data  • List of outcome data that will be collected for participants who discontinue or deviate from intervention protocols | 7-8 |

|  |  | outcome data to be collected for participants who  discontinue or deviate from intervention protocols | • Any plans to record the reasons for:  o Non-adherence (e.g., discontinuation of intervention due to harms versus lack of efficacy)  o Non-retention (withdrawal from trial, lost to follow-up) |  |
| --- | --- | --- | --- | --- |
| Data management | 26 | Plans for data entry, coding,  security, and storage, including any related processes to  promote data quality (e.g.,  double data entry; range checks for data values). Reference to  where details of data  management procedures can be accessed, if not in the  protocol | • Processes for data management, including:  o Data entry and coding, including measures to reduce errors (e.g., double data entry; range checks for data values):  o Data security  o Data storage, including time frame  • Reference to where full information can be found (e.g., Data Management Plan), if not in the protocol | 8-9 |
| Statistical methods | 27a | Statistical methods used to compare groups for primary and secondary outcomes,  including harms | • Statistical methods for each analysis  o Main analysis method for statistical comparison  o Effect measure (e.g., absolute risk) with confidence intervals  o Statistical significance level  o For Bayesian analysis: choices of priors, computational choices, details of any modelling, and effect measure with credible intervals  • For adjusted analyses (if applicable):  o Rationale for adjusted analyses  o List of covariates for adjustment  o Statistical methods  o If both adjusted and non-adjusted analyses are planned, which will be the main analysis  • Methods to account for multiplicity, if applicable  • Reference to the full statistical analysis plan, if a separate document exists | 9-10 |
|  | 27b | Definition of who will be  included in each analysis (e.g., all randomized participants), and in which group | • Who will be included in the primary and other analyses (e.g. all randomized participants with either observed or imputed outcome data)  o Any exclusions due to missing data or other reasons  • Trial group in which participants will be analyzed (e.g., as-randomized) | 9-10 |
|  | 27c | How missing data will be handled in the analysis | For each analysis:  • Assumption about the missing data mechanism (e.g., missing at random), with justification | 9 |

|  |  |  | • How missing data will be handled (e.g., multiple imputation, model-based approaches), with justification |  |
| --- | --- | --- | --- | --- |
|  | 27d | Methods for any additional analyses (e.g., subgroup and sensitivity analyses) | For any planned subgroup analyses:  • Baseline variables to be explored  • Rationale  • Statistical methods (e.g., test of interaction)  • Cut-points and rationale for categorization of continuous baseline variables (if applicable)  For any planned sensitivity analyses:  • Rationale  • Statistical methods | 9 |
| **Methods: Monitoring** | | |  |  |
| Data monitoring committee | 28a | Composition of data monitoring committee (DMC); summary of its role and reporting structure; statement of whether it is  independent from the sponsor and funder; conflicts of interest and reference to where further details about its charter can be found, if not in the protocol.  Alternatively, an explanation of why a DMC is not needed | • Whether a DMC is planned, with rationale  If DMC is planned:  • Composition of DMC  o Size and characteristics of membership (e.g., type of expertise)  o Chair and member names (if known)  • Roles and responsibilities  • Reporting structure  • Method of operation (e.g., meeting format and frequency)  • Degree of independence from those conducting, sponsoring, or funding the trial  • Reference to DMC charter where further details can be found | 8-9 |
|  | 28b | Explanation of any interim  analyses and stopping  guidelines, including who will have access to these interim results and make the final  decision to terminate the trial | Interim analyses:  • When they will be conducted (timing and indications), and by whom  • Statistical methods  • Who will have access to interim results, and whether they will be blinded  Stopping guidelines:  • Any criteria (statistical or non-statistical) that will be used to inform decisions about early stopping or other adaptations (e.g., sample size re-estimation)  • Who will make the decision to continue, stop, or modify the trial | 9-10 |
| Trial monitoring | 29 | Frequency and procedures for monitoring trial conduct. If | • Approach for monitoring (e.g., central, remote, on-site, risk-based)  • Scope of monitoring activities (e.g., type and amount of data at each site)  • Anticipated frequency of monitoring activities | 8-9 |

|  |  | there is no monitoring, give explanation | • Who will be involved in monitoring  • Reference where further details can be found (e.g., monitoring plan)  • If no monitoring is planned, this should be stated with reasons |  |
| --- | --- | --- | --- | --- |
| **Ethics** | | |  |  |
| Research ethics approval | 30 | Plans for seeking research  ethics committee/institutional review board approval | • Plans to obtain research ethics committee/institutional review board approval | 4 |
| Protocol amendments | 31 | Plans for communicating important protocol  modifications to relevant parties | • Process for making protocol amendments, including:  o Decision-making authority for protocol amendments  o How substantive changes will be communicated to relevant parties (e.g., REC/IRBs, trial registries, regulatory agencies) | 4 |
| Consent or assent | 32a | Who will obtain informed  consent or assent from  potential trial participants or authorized proxies, and how | • Role, experience, and training of individuals obtaining consent  • How consent will be obtained from potential participants  • If applicable, how assent will be obtained from paediatric participants who are too young to consent, including:  o How information will be provided to potential participants  o How their understanding and assent will be ascertained  • Any plans to obtain proxy consent from potential adult participants who lack decisional capacity, including:  o Who will determine the individual's decisional capacity  o Any formal capacity instrument to be used  o Any plan for securing informed agreement to continue participation once decisional capacity is regained | Table 1 |
|  | 32b | Additional consent provisions  for collection and use of  participant data and biological specimens in ancillary studies, if applicable | • How consent will be obtained for using participant data and biologic specimens in specified or unspecified ancillary studies  • How the data and specimens will be collected and stored for ancillary studies | Table 1 |
| Confidentiality | 33 | How personal information  about potential and enrolled  participants will be collected,  shared, and maintained in order to protect confidentiality  before, during, and after the trial | • How confidentiality will be preserved when:  o Collecting and maintaining personal information before, during, and after the trial  o Transmitting data to sponsors, co-investigators, and external parties | 8-9 |

| Ancillary and post-trial care | 34 | Provisions, if any, for ancillary and post-trial care, and for  compensation to those who suffer harm from trial  participation | • Any plans to provide or pay for ancillary care during the trial  • Any care or benefits that will be provided to participants or host communities after trial completion  • Any plans to compensate participants for trial-related harms  • If no plans for ancillary and post-trial care, this should be stated with reasons | NA |
| --- | --- | --- | --- | --- |
